# How to prove neglect in the context of the post-mortem examination

**DOI:** 10.1007/s12024-023-00695-2

**Published:** 2023-08-14

**Authors:** L. Lutz, M. F. Klinger, F. Holz, M. A. Verhoff, J. Amendt

**Affiliations:** Institute of Legal Medicine, University Hospital Frankfurt, Goethe-University, Kennedyallee 104, D-60596 Frankfurt Am Main, Germany

**Keywords:** Abuse, Nursing, Myiasis, *Musca domestica*, *Lucilia sericata*

## Abstract

Understanding the causes, extent, and period of neglect is not only a medical but also a forensic task when it comes to legal investigations. In this study, we evaluated 46 autopsied cases where there was clear evidence of physical neglect during the last period of the deceased’s life. The age of the deceased ranged from 21 to 96 years; most of them were female (71.7%). The majority of cases (89.9%) took place in a domestic environment, with partners or relatives providing care. The most frequent post-mortem findings were pressure sores, followed by inflammatory skin changes, and signs of malnutrition and dehydration. Neglect was the cause or co-cause of death in 23% of the cases. More than half of the deceased showed severe contamination of the skin surface by excrement, and in almost 40% of the cases, fly infestation was found. The majority of insects belonged to the group of house flies (Diptera: Muscidae), mainly the common house fly, *Musca domestica*. By analyzing the entomological evidence, it was possible to prove an insect infestation period of at least several days ante-mortem. Since the period of neglect may be relevant in terms of legal proceedings, the present work demonstrates the particular importance of insect traces in providing this evidence. While prosecution and conviction of caregivers remain challenging, it is all the more essential that entomology and legal medicine collaborate on the analysis of findings of neglect.

## Introduction

The term “elder abuse” includes a wide range of acts that intentionally or through neglect result in harm or involve at least the potential for serious harm to an older adult [1]. While abuse includes physical, sexual, or emotional acts, neglect is the refusal or failure by those responsible to provide food, shelter, health care, or protection for a vulnerable elder [2]. The World Health Organization defines the abuse of older people as “an intentional act, or failure to act, by a caregiver or another person in a relationship involving an expectation of trust that causes harm to an adult 60 years and older” [3]. Such misconduct may result in serious physical injuries and long-term psychological consequences, increased risk of nursing home placement, use of emergency services, hospitalization, and premature death [4–6]. However, it is not always the responsibility of others. Often, the neglect can be self-inflicted, because the person concerned is not able or willing to take sufficient care of him/herself for a variety of social, medical, or psychological reasons [7–10]. The phenomenon of neglect and abuse of older people is becoming increasingly important in various disciplines such as medicine and social care [11, 12]. It will intensify in the near future, since the absolute number of older people affected by abuse is projected to increase as the world’s population grows. Already nowadays, the physical neglect characterized by insufficient care, clothing, housing, and hygiene is becoming more and more common not only in nursing homes but also in cases where the victims are cared for in a private setting [13–15]. In determining neglect in such cases, medico-legal examination of the living can help to understand the history of events and a possible criminal background [16, 17]. In cases of death, the autopsy findings and the analysis of ante-mortem data can prove that neglect or abuse had played an important role in the deaths of these persons [18]. Besides the typical physical findings of neglect such as infectious skin diseases, exanthema, ulcerations, immobilization, and pressure ulcers, the deceased often show an overall poor physical appearance, e.g., soiling with excrement over the entire body [1, 16]. In such cases, it is not unusual to find insects on the bodies during their lifetime [19]. Myiasis is defined as “the infestation of live humans and vertebrate animals with dipterous larvae, which, at least for a certain period, feed on the host’s dead or living tissue, liquid body substances, or ingested food” [20, 21]. Typical species in such cases often belong to the blow flies (Diptera: Calliphoridae), flesh flies (Diptera: Sarcophagidae), house flies and their relatives (Diptera: Muscidae), and lesser house flies (Diptera: Fanniidae). While the first two families mainly prefer already developed sores and necrotic tissue, e.g., of the diabetic foot [7, 19], muscids are found in classic situations of neglect, where excrement and other spoilage play an important role [22, 23]. Species of this group are very prevalent in neglect settings due to their strong synanthropic behavior [24], and their larvae develop primarily in compost, food scraps, and excrement. As mentioned already, insect colonization is not only another physical characteristic of neglect but can act as an important tool for forensic examinations, such as establishing the timeline of events and understanding the history of the specific neglect [19, 25]. The impetus for the current study was a 2014 death investigation that highlighted the strength of interdisciplinary work between legal medicine and forensic entomology to prove neglect.

### Case study

In September 2014, the body of a 64-year-old woman was found in the living room of her apartment after her grandson called the ambulance service and the police. She lived in a two-room flat with her daughter and two grandsons, who were responsible for her care. The body was found in supine position on the couch, wearing just a T-shirt. After removing the body, a heavy larval infestation and large amounts of excrement were found on the couch. Overall, the entire flat was in a poor state of cleanliness, being dirty, littered, and neglected and according to the first responders, it “smelled beastly.” However, the living room appeared to have been cleaned up before the police arrived. Based on the findings, the criminal investigation department and the prosecutor’s office requested an autopsy to determine the cause of death and quantify the influence of the negligence. Due to the obvious signs of physical neglect, all three caregivers from the family were reported to the police. The autopsy revealed clear signs of physical neglect, such as inflammatory ulcerated skin lesions on the buttocks and hips; overall poor personal hygiene with uncut, claw-like toenails and fingernails; and multiple pressure ulcers on the back and thighs. The cause of death was a bilateral pneumonia. In addition to the medical/physical findings, entomological signs of neglect, i.e., larval infestation in the pressure ulcers, were found. The entomological evidence was sampled and analyzed, and the period of insect infestation was estimated based on insect ID, temperature, and published developmental data. The results revealed an infestation by the housefly *Musca domestica* (Diptera: Muscidae). The oldest developmental stages in the present case were full puparia. Based on an assumed temperature of 35 °C (due to the development close to the body), a minimum development period of 3–4 days could be assumed until this stage was reached, indicating an insect infestation while the woman was still alive. To analyze the incidence of similar cases of physical neglect in the greater area of Frankfurt am Main, Germany, and to highlight the strength of interdisciplinary analysis demonstrated here (entomology and legal medicine), a retrospective study of similar cases was conducted.

## Material and methods

### Data basis

We evaluated 46 cases from 1994 to 2021 in which there was evidence of physical neglect during the last period of the deceased’s life. Forty-one cases were autopsied and analyzed at the Institute of Legal Medicine Frankfurt am Main; the remaining five cases originated from other German Institutes of Legal Medicine. Where a detailed case history was available, the insects were sampled, and based on this, at least one of the authors prepared an entomological report. In two cases, i.e., insect infestation of tracheostomata, the person did not die, and thus, only entomological reports were requested to estimate the period of insect infestation. For each case, information was noted, if available, on the following:

- Date (day, month, year) of the discovery and the autopsy
- Sex and age of the deceased
- Maximum post-mortem interval (PMI_max_), based on the time of last seen alive
- Place of discovery (private, nursing home, or hospital), including particulars such as the state of cleanliness and the use of the room
- Person who were responsible for the care of the deceased
- Manner (natural, unnatural, unclear) and cause of death
- Police investigation (interviews with the caregivers, psychological assessment of their mental state)
- Prosecution (court proceedings, convictions)

### Physical classification

The physical neglect of the deceased was categorized based on Püschel [16]. First, we assessed if the neglect was intentional (active), i.e., withholding items of necessity, such as utensils for hygiene, food, or medication, or unintentional (passive), i.e., the neglect was caused by the individual’s or caregiver’s physical or psychological impairment. To evaluate and classify the pattern and grade of neglect, we investigated these physical findings:

- Marasmus (severe loss of body weight due to caloric/energy deficiency)
- Exsiccosis (dry fatty tissue, skin folds remaining elevated, dry and scabby tongue)
- Superinfections and decubitus ulcers
- Infectious skin diseases: exanthema, ulcerations
- Immobilization: contraction of joints
- Starvation, dehydration, malnutrition: small intestine, hard dry stool in the colon
- Poor dental hygiene
- Soiling with excrement over the body
- Uncut, claw-like toenails and fingernails
- Matted head hair
- Insect infestation: fleas or lice, different developmental stages of necrophagous and necrophilous flies (i.e., eggs, larvae, full or empty puparia, adults)

### Entomological findings

Entomological evidence, i.e., juvenile and/or adult stages, or remains of insects, was sampled. Adult flies (at – 20 °C) as well as larvae and pupae (with almost boiling water) were killed and then stored in 96% ethanol, while insect remains such as empty puparia were stored directly in 96% ethanol. In some cases, larvae and pupae were reared in the laboratory under constant temperatures until the emergence of the adult flies and the times for reaching specific developmental landmarks (post-feeding, pupariation, adult emergence) were noted. Species identification was performed based on morphological characters with the current systematic literature [26, 27]. In 10.8% of the cases (*n* = 5), an entomological report was requested by the prosecution. In cases with a question about the time of death and neglect based on the insect ID, larval length and/or developmental landmarks and the reconstructed temperature of the death scene, the period of insect infestation was estimated. This was followed by age determination with the help of reference literature such as [28–30]. The results were compared with the PMI_max_ and pathological findings to highlight possible discrepancies between time of death and that of the first insect colonization on the body.

### Legal proceedings

Access to the case files was requested from the Prosecutor General’s Office. In cases where the file situation (previous history, investigation data, interviews with the caregivers) made it possible, the question of responsibility (who was the person responsible for the care) was answered and it was clarified to what extent there were criminal investigations afterwards and with what results, such as court proceedings and convictions.

## Results

**Fig. 1:**
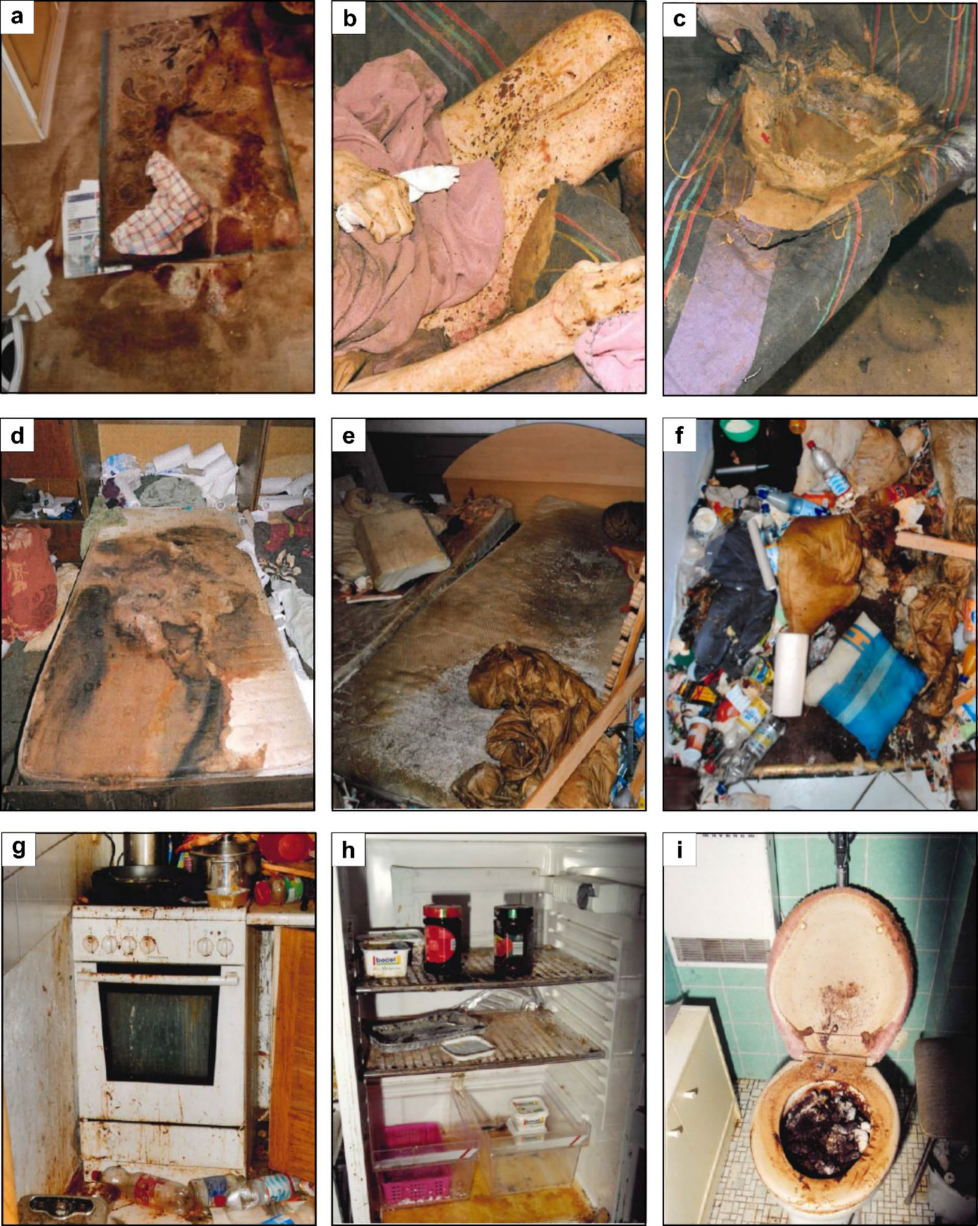
Examples of typical finding situations of deceased who showed signs of physical neglect. **a** Carpet on which a 71-year-old woman was cared for by her husband for 3 weeks after fracturing her femur. **b**, **c** Underlayment (mattress) on which an 83-year-old woman was cared for by her son. **d** Underlayment (mattress) in shared marital bed on which a 77-year-old woman was cared for by her husband and son. **e–g** Apartment and pad where a 49-year-old woman was cared for by her husband and son. **h**, **i** Apartment of a 71-year-old woman who was cared for by her daughter in the shared apartment

A total of 46 cases from 1994 to 2021 were analyzed (Table 1), 89.2% of which were autopsies from the Institute of Legal Medicine Frankfurt and 10.8% were just entomological reports from other German Institutes of Legal Medicine. The age of the deceased ranged from 21 to 96 years and most were female (71.7%). In the majority of cases (89.9%), the deceased were “cared for” in a domestic environment, e.g., their own flats, with a partner or relatives (children, grandchildren) providing care (Table 1). For many cases (78.2%), we were able to determine the category (passive, active) of neglect. In 75% of these cases, the neglect was unintentional due to the individual’s or caregiver’s physical or psychological impairment. In 25% of cases, the neglect was intentional and the caregiver, almost exclusively nursing services, had withheld necessary items such as hygiene items, food, or medications. In cases where the persons died in their own homes (~ 80%), the ambulance and/or police were called by the caregivers, minutes to hours after the death of the person. Upon arrival, the rescue services found in most of the cases a person in a neglected physical condition, who lived and died in squalid, unhygienic, and generally desolate conditions. Figure 1 illustrates some of these situations.

**Table 1:** Information on 46 analyzed cases of physical neglect from 1994 to 2021

**Cases with signs of physical neglect (1994–2021)**	
**Analyzed cases**	46
Autopsies (Institute of Legal Medicine)	89.2%
Entomological report	10.8%
**Care situation**	
Nursing home	10.8%
Domestic environment	89.2%
**Caregiver**	
Relatives (spouse, children, grandchildren)	87.0%
Nursing service (ambulant)	13.0%
**Category of neglect**	
Information available	78.2%
Thereof intentional (active)	25%
Thereof unintentional (passive)	75%
**Criminal pursuit**	
Investigation proceedings	71.7%
Thereof court proceedings	24.2%
Thereof sentencing	25%*
**Information deceased**	
Female	71.7%
Male	28.3%
Age (minimum)	21**
Age (median)	74
Age (maximum)	96

^*^Court documents are consulted in only 37% of cases; ^**^Case of insect colonization of a tracheostoma of a young man

### Physical classification

**Fig. 2:**
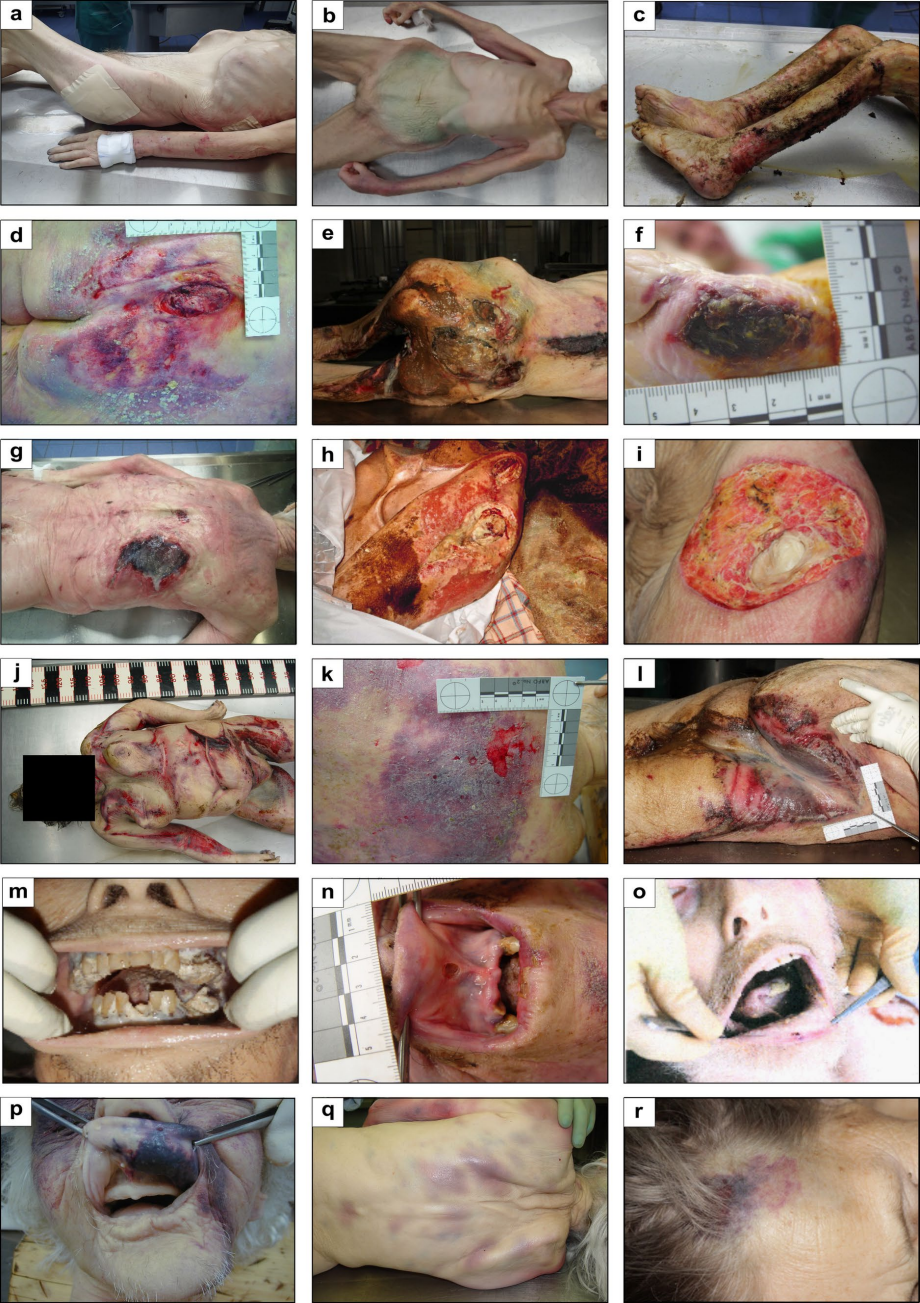
Medical findings of physical neglect (permanent). Examples of **a**–**c** malnutrition, **d**–**i** pressure ulcers, **j**-**l** inflammatory skin lesions, **m**–**o** poor/catastrophic dental hygiene, and **p**–**r** hematomas

**Fig. 3:**
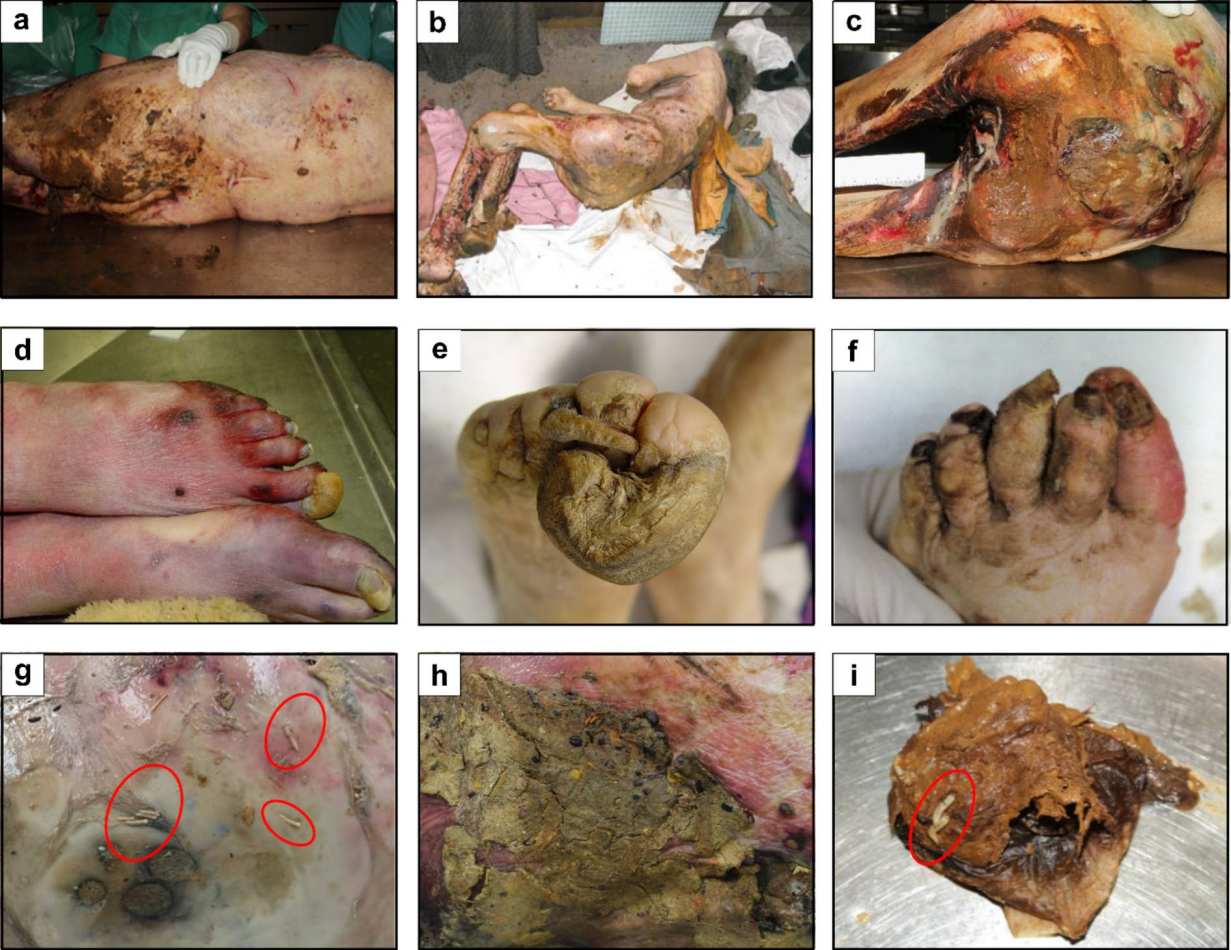
Forensic and entomological findings of physical neglect (non-permanent). **a**–**c** Contamination by excrement (feces, urine); **d**–**f** claw-like toenails and fingernails; **g**–**i** insect colonization (maggots, pupae, empty puparial cases)

During the post-mortem examination on site as well as in the autopsy, the physical neglect of the deceased was proven in most cases by the medical examinations (Table 2). The most frequent findings (76.1%) were pressure sores (decubitus ulcers), some of which had inflammatory changes and in extreme cases extended to the bone (Fig. 2d–i), followed by inflammatory skin changes (63.0%, Fig. 2j–l), a poor to abysmal dental hygiene (37.0%, Fig. 2m–o), and signs of malnutrition and dehydration (Fig. 2a–c). In addition, more than half of all the deceased showed severe contamination of the body by excrement (Fig. 3a–c), besides other typical findings such as claw-like, uncut toenails and fingernails (Fig. 3d–f) and matted hair. Despite the sometimes severe physical findings, neglect was the cause of death or co-cause of death in only 23% of the cases. In these cases, the cardiovascular failure was due to sepsis from the decubitus ulcer.

**Table 2:** Information on the forensic medical findings of physical neglect

Physical finding	Cases
Marasmus	15.2%
Exsiccosis	30.4%
Decubitus ulcers	76.1%
Buttocks	63.0%
Heels	28.0%
Shoulder	26.0%
Hips	30.0%
Other	11.0%
Infectious skin disease	63.0%
Immobilization	26.1%
Starvation, dehydration, malnutrition	8.6%
Poor dental hygiene	37.0%
Bruises	21.7%
Soiling with excrements over the entire body	56.5%
Uncut, claw-like toenails and fingernails	54.3%
Matted hair	23.9%
Insect infestation	36.9%
Fleas, lice	4.3%
Necrophagous flies	36.9%

### Entomological findings

**Fig. 4:**
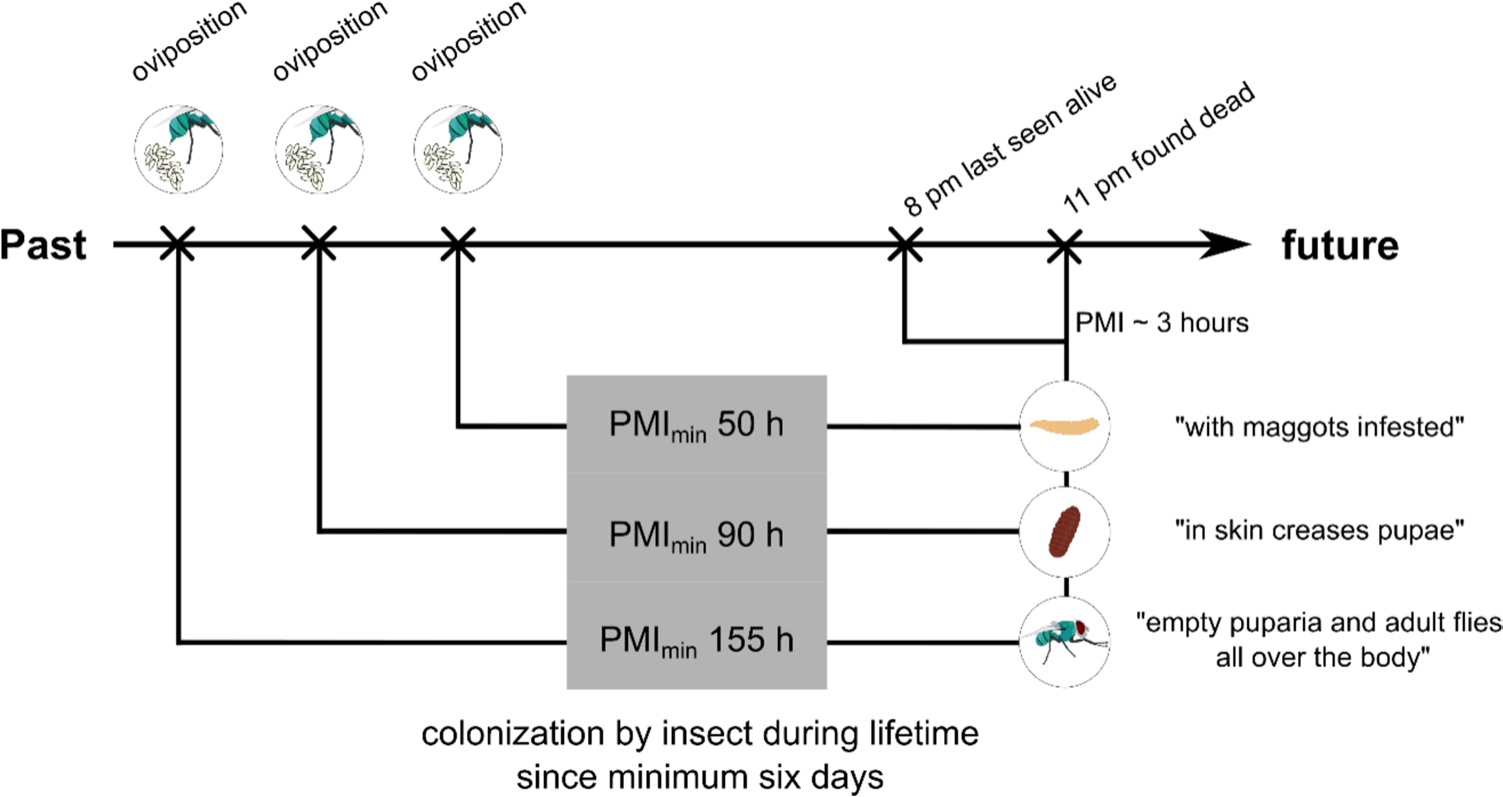
Graphical representation of the estimation of the period of infestation using entomological traces in a case of neglect of a 77-year-old woman. The PMI_max_ based on the information provided by the care-giving relatives was approximately 3 h. The determination of the time of insect colonization using larvae and empty and full puparia as well as adult flies of *Musca domestica* and a temperature of 35 °C resulted in a period of 50–155 h, depending on the stage of development used. The PMI_max_ and the period of insect colonization are in discrepancy to each other, indicating an insect colonization on the body of the female before her death

In almost 40% of the cases, insect infestation was found in addition to the other findings of physical neglect. The infestation included in some cases only a few small fly larvae up to a mass infestation by mature larvae, with full and/or empty fly puparia. The conditions were, e.g., described as follows: “in skin folds pupae and puparia,” “feces and urine were found on the sofa, also maggots,” “maggots visible on the skin,” and “few puparia and isolated maggots.” Only a few cases showed blow fly infestation, usually represented by the species *Lucilia sericata* (Diptera: Calliphoridae), while the majority of insects belonged to the group of house flies (Diptera: Muscidae). Many species of this group have developed a close relationship with humans and their settlements, called synanthrophy, and the degree and nature of this relationship depend on the fly species and the geographic and climatic specifications, as well as the characteristics of the human environment [24, 31, 32]. In addition, their larvae develop primarily in compost, food scraps, and excrement. The presence of these flies in cases of neglect can be explained by the typical situation in which many of the deceased were found (“swimming in excrement”). Insect colonization is not only another physical characteristic of neglect but can act as an important tool for temporal classification of neglect. By analyzing the entomological evidence on the corpse, it was possible to prove in all cases that the insect infestation was already present during the lifetime of the deceased, i.e., the time of colonization was before the time of death, exceeding the PMI_max_ up to a week. Figure 4 illustrates a case in which insect traces on the corpse could prove an insect infestation started at least 6 days ante-mortem.

### Legal proceedings

In 70% of the cases (Table 1), an investigation was initiated and criminal charges were filed against the caregivers. In the majority of cases, however, the proceedings were discontinued as there was no reasonable suspicion of deliberate neglect or third-party misconduct could not be proven. In most cases, this was due to the “emotional overload” of the caregivers. There were court proceedings in 24.2% of the cases in which preliminary proceedings had been initiated beforehand. Only two cases resulted in a conviction. In the first case, the neglect of a 78-year-old woman, the caring daughter was sentenced to 21-month probation for abuse of a ward. In the second case, the neglect of a 94-year-old woman, the caring daughter and grandson were sentenced to 24- and 18-month probation, respectively, for abuse of a ward and bodily injury with fatality. According to the court’s findings, the daughter and grandson had not cared for the woman with dementia for months, although they would have been legally obligated to do so. In the end, the emotional overload of the relatives led to the victim’s death.

## Discussion

The medical and entomological assessment of 46 neglected bodies revealed appalling care deficiencies, but also the need for analysis of entomological evidence in such cases, as insects can act as important indicators of neglect in the prosecution proceedings.

With regard to the gender of victims of elder abuse, including psychological, financial, and sexual abuse as well as abandonment and neglect, the literature indicates that women are at greater risk [33, 34]. Similarly, in our study, ~ 72% of the neglected persons were female. Drommi et al. (2021) [13] investigated not just the question of the victims gender but also of the perpetrators. Their study showed that a majority of cases of abuse are perpetrated by men: indeed, of 276 people, who were charged with abuse, 178 were men and 98 women. In our study, we found similar results, with twice as many men as women being perpetrators or neglecters. However, the focus of Drommi et al. [13] study differed from ours—they analyzed cases prosecuted in Genoa (Italy), of proven neglect of people who were still living and included cases not only typical caregiver neglect but also of domestic violence, assault, and even financial exploitation. The caregiving men in our study were either partners or sons of the neglected women. In one case, even the husband and the common son acted together in the neglect of their wife/mother. This degree of familial association between victim and perpetrator is matched by the fact that the most common site of abuse is the victim’s own home (where victim and perpetrator usually, but not always, lived together).

Residential facilities appear to be safer than the domestic environment, not least because of the introduction of modern systems for monitoring staff and accrediting the facility. Indeed, of the 46 cases, only three were found in a professional care setting. Two of them showed unusual findings of neglect, as they were coma patients whose tracheostoma was colonized by insects. In both cases, the infestation was caused by the blow fly *Lucilia sericata* (Meigen, 1826), which had only been on the tracheostoma for 1–2 days and were discovered when the tube was changed, a similar infestation to nosocomial infestations reported in hospital care settings [20, 35, 36]. This phenomenon has already been reported in the literature but more often in countries with warm climate such as Malaysia or India [35, 37, 38]. While *Lucilia sericata* is the most common myiasis causing blow fly species in Europe [19, 39], it can be used beneficially in wound treatments to break down necrotic tissue and combat multi-resistant bacteria [40]. In both tracheostoma cases of our study, the colonization was favored by an unusually warm period before and during the time of infestation. On the one hand, this illustrates the thermophilous behavior of *L. sericata* [41], but at the same time, it shows that even in very sensitive and vigilant care units, colonization by flies cannot be categorically ruled out, e.g., as windows are opened for ventilation. This is also shown by the infrequent but regular requests for the assessment of myiasis by various hospitals (unpublished data). The third case in a professional care facility was of a 96-year-old woman, and it illustrates the difficulties of assigning responsibility in such complex caregiving situations, where no single person is 100% responsible for the neglect and, potentially, fatal outcome. The woman was initially cared for at her home, where she fell and was eventually moved to a nursing home due to the difficult care situation. Six weeks later, she was admitted to a hospital, where she died 3 days later as a result of extensive pneumonia in both lungs with concomitant significant cardiac damage. Extensive pressure ulceration on the coccyx and an initial decubitus ulcer on the right heel were noted. Further microbial investigations would have been necessary to determine whether the decubitus ulcers could have been considered an entry point for bacterial colonization and thus as the origin of the pneumonia found.

Almost all of the cases studied here occurred in a domestic environment and took place indoors. Only one case involved a homeless person. This 44-year-old man was infested with fly larvae during his lifetime, a common phenomenon for homeless people but still rarely the focus of scientific research of neglect [42, 43]. It might be surprising at the first glance, that homeless people are not more represented in our study, because a higher rate of neglect, including insect infestation of open wounds or infestation by common ectoparasites of poverty, such as lice, bed bugs,or fleas [44], might be expected in the homeless community [45]. Research studies of deceased homeless persons from German forensic medical institutes evaluated the proportion of neglected persons and reported up to 25% [46] and respectively 2.7% with larval infestation, but no further entomological examination [47]. In such cases, we are usually dealing with self-neglect while our current study focuses on neglect by caregivers.

The majority of cases in our study happened in a domestic environment, where the neglect of a person, including insect infestation and its extent, can remain hidden, even more so if the caregiver makes an effort to hide it from the outside world. Therefore, it is not surprising that 80% of the neglected persons died unnoticed at home without first receiving medical attention. As death approaches slowly and is accompanied by a steady, perhaps gradually increasing neglect, the scenes at the time of discovery of the bodies are often unexpectedly dramatic for the first attenders. In this study, many victims of neglect were found lying in their own excrement, according to on-site medical personnel, in “disgusting and extremely inhumane situations.” In extreme cases, the mattresses were “littered with feces and dried insect larvae.” It seems hard for a normal person not to imagine that these situations were unbearable, not only for the person being cared for but also for the caregiver. However, daily exposure and adaptation to such circumstances may play an important psychological role. In particular, the mental and physical health of the caregiver is an important factor in assessing whether the neglect was intentional or unintentional. In the majority (75%) of the cases studied, the caregiver was simply emotionally and mentally overwhelmed with caring for their loved one, which was impressively demonstrated in two cases where the caregiver committed or attempted suicide after the death of the person they cared for. However, neglect can be intentional, and in these cases (25%), years of aversion (to the point of hatred), or in cases of neglect by nursing services, lack of conscientiousness, and empathy, also appear to play an important, almost driving role. But it is also common that, against the will of the caregiver, the person in need of care refuses help from third parties and forces the caregiver not to get anyone to help. In one case, for example, the wife refused any medical care and prevented the caregiving husband from calling a doctor.

The medical findings in the majority of cases were typical pressure sores (decubitus), sometimes with inflammatory changes, and in extreme cases, the lesions extended to the bone. Associated were poor to appalling hygiene standards, with more than half of all the deceased showing severe contamination of the body surface with excrement. The latter is closely linked to the most important entomological finding of the study, the prevalence of the house fly *Musca domestica*. Flies infested almost half of the victims of the present study and were present for different periods and in different stages of development. Very often blow flies play an important role in the colonization of the neglected living humans, due to the presence of necrotic tissue, in decubitus ulcers and other lesions [19]. However, probably, the overwhelming fly attractant in our cases was fecal material, which is extremely attractive to the house fly *M. domestica*. Even if the presence of insects is understandably classified by most as disgusting and to be avoided at all costs, their presence can be used in the context of the legal processing of the findings. Insects have been routinely used in forensic science for many years as a tool to narrow down the time of death [48]. For this purpose, the temperature-dependent and species-specific aging process of the juvenile stages (egg, larva, pupa) is calculated. In cases of neglect, this same insect age provides the time of colonization and thus provides a minimum period of neglect rather than death. Thus, it is important in each death investigation to bear in mind that entomological samples are not always physical evidence of a post-mortem infestation. Depending on the temperature and species, time periods of up to 14 days can be determined to the day. It is surprising that in numerous studies, insect infestation is detected without performing species identification of the available samples or using the presence of insects to delimit periods of neglect. Our data clearly show that this can be a relatively straightforward analysis and should be a standard procedure in the future to answer the question of insect colonization before death.

The question of whether an entomological examination can provide information about possible insect-related infections is also frequently raised by the court. In cases of neglect, it seems difficult to prove secondary transmission of pathogens by insects with certainty, because the pathogen load on insects appears to be too low to cause sanitary problems, yet it cannot be ruled out [49, 50]. However, there are many sources of infection besides insects. For example, many skin wounds in these situations would be colonized by human skin flora and also bacteria associated with excrement.

Despite the severe physical findings, neglect could only be proven as the cause of death or as a contributory cause of death in 23% of the 46 cases. In the other cases, the focus was mostly on the underlying disease that led to the need for care. This explains in part the low number of further prosecutorial investigations. On the other hand, for a criminal conviction, the neglectful actions must be attributable to a specific person(s). In the 23 cases in which the relevance of neglect to death was (partly) affirmed, sepsis was present as a result of the pressure ulcer. At the same time, the social and family conditions were very complex and responsibilities were very difficult or impossible to determine due to the personal involvement of close family members. They were often overburdened and psychologically severely affected by the situation. Therefore, it is not surprising that in only two cases did neglect result in a conviction of the caregiver despite the compelling findings of neglect.

With the progressive aging of the world’s population, it is necessary to increase our knowledge of the characteristics of neglect and its associated mechanisms, to undertake appropriate prevention and, where this fails, to ensure rapid identification and effective treatment of the phenomenon. However, even in the worst-case scenario, i.e., the death of the patient under inhumane conditions, it is part of good practice to carry out professional documentation and forensically clean processing. Our study is an example of what such a process and assessment could look like.

## Key points

1. Determining the causes, extent, and duration of neglect is an important forensic task.
2. Forty-six cases with clear evidence of physical neglect in the last period of the deceased’s life were analyzed.
3. Neglect was the cause or co-cause of death in 23% of the cases.
4. Insect infestations, mainly house flies, were found in almost 40% of cases, providing evidence of insect infestation for at least several days ante-mortem.

## Data Availability

The data that support the findings of this study are available from the corresponding author upon reasonable request.
